# Interactive Design of Business English Learning Resources Based on EDIPT Multimodal Model

**DOI:** 10.1155/2022/1264847

**Published:** 2022-09-08

**Authors:** Xiaomei Yang, Shi Qi

**Affiliations:** Fundamentals Department, Luxun Academy of Fine Arts, Shenyang 110816, Liaoning, China

## Abstract

Aiming at the problem that online video learning resources of business English are scattered and the learners are inefficient in acquiring learning resources, this paper designed a business English learning system based on the EDIPT model. In addition, aiming at the problem of multifeature fusion between low-level features and high-level semantic features in video scenes, this paper proposes a multi-modal video scene segmentation algorithm based on a deep network. By minimizing the square sum of distances in the time period, the shots are clustered, and finally, the semantic scene is obtained. The experimental results show that the algorithm has good performance in classification accuracy and can effectively segment video scenes, which is helpful for users to improve their comprehensive business English skills.

## 1. Introduction

Education makes human knowledge and civilization spread, and it is an important force to promote the development of human knowledge and civilization. With the development of science and technology and the increase of knowledge, the society needs more advanced and effective means of education. Using the network to spread knowledge is an effective way to spread knowledge. Among them, business English is an application-oriented major that teachers need to pay attention to the exercise of students' English, especially oral communication, so it is very important to build a good English learning environment [1, 2]. However, many colleges and universities are lack of a good business English learning environment, resulting in students cannot get effective English training. Normally, business English students in the development of oral practice are carried out in the simulation of business activities, the lack of daily teaching of business English oral exercise, so that students' oral English level cannot be effectively exercised and then affect the students' English level.

In the teaching design, the EDIPT design thinking model is widely recognized in the field of education from the perspective of students. It is used to guide teaching practice and is conducive to the development of students' innovation ability and design thinking. Through the implementation of the design thinking process, Lin changed the current situation of single information technology works of junior high school students and provided a teaching model and activity design suitable for junior high school [3]. Yu introduced a foreign typical EDIPT design thinking model into a scratch classroom in primary school, guided scratch teaching according to the operation process of the EDIPT design thinking model, and designed learning activities to improve students' design thinking ability [4]. Design thinking is not only used to guide classroom teaching but also applied to practical teaching by many educational institutions outside school.

In addition, online video learning is an effective means for students to exercise their business English application ability. But at present, the retrieval of teaching video still relies on the TBVR form, which has the following problems [5, 6]. Firstly, the manual annotation only represents the staff's personal views on the video, which is too subjective and difficult to cover each person's grasp of the different focuses of the video; secondly, manual tagging requires staff to make a brief summary after watching the video, but in the face of the explosive growth of massive video data, the time and labor cost of tagging are difficult to estimate; thirdly, the content in the video is abundant, so it is difficult to summarize it with simple words or phrases. For the above-given reasons, TBVR is not conducive to users to quickly find their interesting teaching video clips and knowledge points, and it is difficult to meet the needs of users. The semantic information based on a single modal analysis is always limited. While combining two or more modes for multimodal feature semantic analysis can obtain more abundant video semantic information, which is an effective method to extract video content.

Multimodal theory refers to the phenomenon that various senses interact with each other through language, image, sound, animation, and other elements [7]. However, multimodality can strengthen the communication of verbal meaning, and learners can effectively understand multiple knowledge signals. Cross thought that video information can promote the understanding of business English materials [8], In particular, multimodal theory video resources are represented by images, sounds, and languages. Compared with single-mode learning resources, audio-visual learning resources can reduce the difficulty of business English learning. Most of the users' information comes from reading, listening, and writing in a business environment. A Révalo found that multimodal guidance can better promote learners' understanding of business English. Business English learning emphasizes scenario simulation and pays attention to the cultivation of communicative competence. In addition, the introduction of multimodal theory has a certain theoretical basis for the integration of business English videos [9].

Therefore, this paper extracts and analyzes the video learning resources of business English, introduces the EDIPT thinking model, and designs a business English learning system integrating multimodal information such as text, graphics, audio-visual, animation, and so on, so as to improve the comprehensive business English skills of users with different learning styles.

## 2. Design of Business English Learning System Based on EDIPT

### 2.1. EDIPT Design Thinking

**Figure 1:**
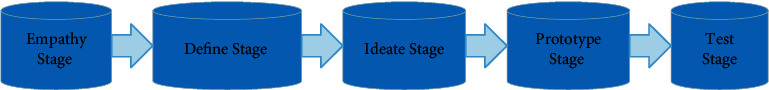
EDIPT design thinking model.

The EDIPT design thinking model consists of five stages that can jump through the cycle: empathy, problem definition, conception, prototyping, and testing, as shown in Figure 1. Each stage includes stage objectives, implementation principles, specific methods, and tools. The specific stages and implementation process are as follows [10, 11].

The specific implementation process of the empathy stage: learners use What? How? Why? Empathy map, situational story method, and other tools, in-depth understanding of the user's environment, in-depth mining of the user's inner activities, to provide a foundation for targeted solutions. The specific implementation process of defining stage: learners use the POV problem definition method to state factual problems as operational problems, using the method of “how might we,” we can ask questions in an open way, decompose and think about the problems, and focus on innovative problems. In the stage of ideating, we can use the scaffold table to think about problems from the seven directions provided in the scamper table or from some selected directions and initially form the problem solution. The realization methods of the prototype stage include sketch drawing, pattern making, and demo design. The specific implementation process: learners can use the simplest tools such as paper and pen to draw sketches, show their own or group ideas, and achieve the goal of expressing ideas, presenting solutions, and quickly realizing creativity. The specific implementation process of the test: learners can use the “feedback capture grid graph” tool to collect and integrate user feedback information, sort out the highlights and problems of the prototype from the collected information, obtain constructive suggestions and further improvement ideas, so as to promote the generation of the best solution.

### 2.2. Teaching Model Design

The whole teaching model includes teaching process design, teaching evaluation design, teaching evaluation design, and teaching feedback design.

The learning mechanism is linear; learners need to complete all learning tasks, and then they can unlock the next video learning. Taking single video teaching as a cycle, the teaching process of this system belongs to cyclic teaching. In addition, learners complete the business English learning module in turn according to the set task objectives. In a single learning cycle, learners acquire business knowledge, improve oral communication, and strengthen other comprehensive business English skills. Video teaching is divided into seven indicators, namely, visual, listening, reading, speaking, testing, translation, and writing, to assess the teaching results. While each teaching task is set with a corresponding score.

### 2.3. Functional Design

**Figure 2:**
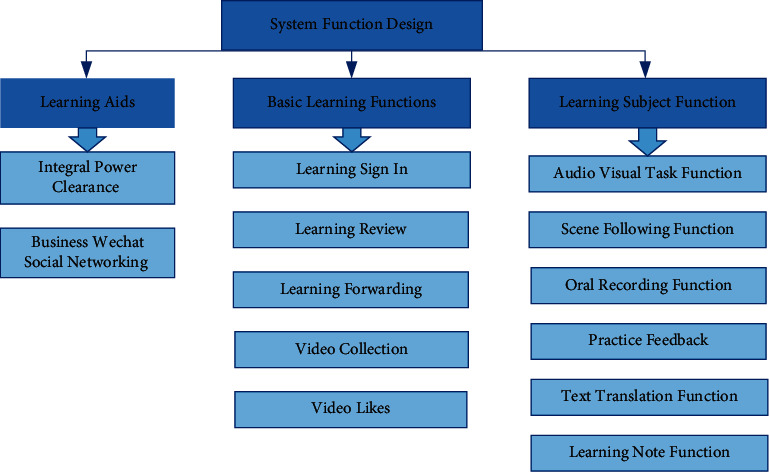
System function design.

By integrating high-quality business English video learning resources, users can improve the efficiency of acquiring learning resources, improve the learners' comprehensive learning ability of business English, create a microbusiness communication platform, and create a business communication circle. The business logic function of the system is divided into three functional modules: basic learning function, learning the main function, and learning auxiliary function. The main function of learning runs through the whole process of video learning and is the core function of the small program. The specific function design is shown in Figure 2.

#### 2.3.1. Basic Learning Function

The basic learning functions include learning check-in, learning review, learning forwarding, video collection, and video like. Check-in design is a basic function commonly used by users, where learners get bonus points for daily check-in, and the points obtained by check-in are converted into scores in equal quantities. Users judge their current learning needs through video reviews, and their diagnostic learning evaluation strengthens their reflection on video learning.

#### 2.3.2. Learning Main Function

The main functions of learning include audio-visual task function, scene following function, oral recording function, practice feedback function, text translation function, and learning note taking function. In the whole learning process, the task-driven teaching method promotes learners to complete video learning. For the key parts of the video content, blank out randomly and fill in the blanks with words. Subtitles are not set in the audio-visual task link. Text translation is attached in the learning link, and the learning note function is added.

#### 2.3.3. Learning Auxiliary Function

The learning assistance function includes two parts: one is to help customs clearance with points and the other is to help the business microgroup to socialize. Redeem the points, add the points to the score, regenerate the score report, view the rating level, help the customs clearance, and unlock the next video learning. The score values of different task modules are recorded in the score report, and the video learning score must be greater than or equal to 90 points to pass the test.

### 2.4. Design of Data Flow

**Figure 3:**
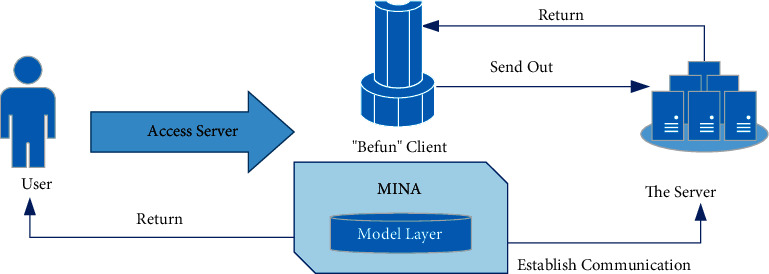
Design of data flow.

Because the system mainly uses user data and video data, the whole process includes three kinds of data flow. The first is that the user sends the behavior request to the system, and the system returns the processing results in the small control layer. The second way is to get the current data information, but it does not involve the change of database information, so it needs to access the background server, where the user sends the access request to the client. After receiving the request, the client realizes C/S communication with the server. Finally, the server returns the business logic processing results to the client, and the client presents it to the user in the form of a page. The third one involves updating the data table information, where the user requests to update or query the data, and the client sends the event request to the server. During the process, the database server program will listen to the network request events, realize the communication between the model layer and the server by passing parameters, and update the information after data proofreading and validation, as shown in Figure 3.

## 3. Multimodal Video Scene Segmentation Algorithm

### 3.1. Overall Design

**Figure 4:**
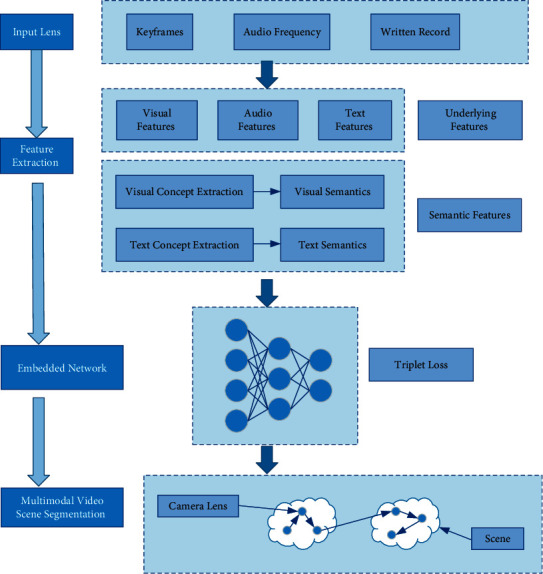
Overall algorithm framework.

Because the above-given system is aimed at video learning resources, the semantic information based on single modal analysis is always limited. While combining two modes or more to carry out multimodal feature semantic analysis can obtain more abundant video semantic information, which is an effective method to extract video content. Therefore, the multimodal deep network method is adopted, where the video scene segmentation task is treated as a supervised time constrained clustering problem. Firstly, rich underlying features and semantic features are extracted from each shot; secondly, in order to obtain the similarity measure between shot features, these features are embedded in Euclidean space; finally, the optimal scene boundary is detected by minimizing the sum of squares of distances in the time period and use a penalty to automatically select the number of scenes. The overall framework is shown in Figure 4.

### 3.2. Embed Deep Networks

Considering that the shots in the same scene usually have the same content in the video stream, the scene segmentation problem is also considered as the problem of grouping adjacent shots together, which is to maximize the semantic consistency of similar shots. In order to reflect the semantic similarity, it is necessary to calculate the distance between lens feature vectors *X*, so an embedding function *φ*(*X*) is constructed, which can map a lens feature vector to the space where Euclidean distance has the required semantic properties. The distance matrix is

(1) $$\begin{array}{c} \left[\varphi \left(X_{i}\right)-\varphi {\left(X_{j}\right)}^{2}\right]=\left[1-\alpha_{i,j}\right], \end{array}$$

where *α*_*i*,*j*_ is a binary function, indicating whether the scene *X*_*i*_ and *tX*_*j*_ belong to the same scene, *i*, *j*=1,2, ⋯, *N*.

The embedded function *φ*(·) makes the shots of a particular scene *X*_*i*_ closer to all shots of the same scene *X*_*i*_^+^, rather than any other shots of any other scene *X*_*i*_^−^, so that the constraint can be carried out.

(2) $$\begin{array}{c} \varphi \left(X_{i}\right)-\varphi {\left(X_{i}^{+}\right)}^{2}<\varphi \left(X_{i}\right)-\varphi {\left(X_{i}^{-}\right)}^{2}. \end{array}$$

In order to improve the embedding ability, a triple depth network is designed, which is composed of three basic networks, where the same parameters are shared, and each parameter takes the scene descriptor as the input and calculates the required embedding function. Train the network loss of Triplet (*X*_*i*_, *X*_*i*_^+^, *X*_*i*_^−^) through the Triplet loss function, and Hinge loss of Triplet is defined as follows:

(3) $$\begin{array}{c} L_{i}\left(w,\theta \right)=m\left(0,\varphi \left(X_{i}\right)-\varphi {\left(X_{i}^{+}\right)}^{2}+\left(1-\varphi \left(X_{i}\right)-\varphi {\left(X_{i}^{-}\right)}^{2}\right)\right), \end{array}$$

where *w* is the network weight; *θ* is the deviation. The total loss of *N* triples is given by the average loss of each triplet plus the *L*_2_ regular term of the network weight to reduce overcompensation.

Therefore, the total loss of N triples can be defined as follows:

(4) $$\begin{array}{c} L\left(w,\theta \right)=\frac{5\times 10^{-5}}{2}{\left\|w\right\|}^{2}+\frac{1}{N}\sum_{i=1}^{N}L_{i}\left(w,\theta \right). \end{array}$$

### 3.3. Multimodal Scene Segmentation

Because scenes need to be continuous in time, scenes with similar semantic content but far away in time should be distinguished. The task of video scene segmentation is treated as a supervised time constrained clustering problem; secondly, in order to obtain the similarity measure between shot features, these features are embedded in Euclidean space; finally, the optimal scene boundary is detected by minimizing the sum of squares of distances in the time period, and a penalty term is used to automatically select the number of scenes.

In order to obtain scene segmentation of video, shots are required to be as semantically consistent as possible. Inspired by K-means, cluster homogeneity can be described by the sum of square distances between cluster elements and their centroids, which is called within cells sum of squares (WSS) [12]. Therefore, the reasonable goal is to minimize the sum of squares within a group, that is, the sum of all WSS. While only minimizing the sum of squares within a group will lead to a trivial solution of only one scene in each sequence. Therefore, it is necessary to add penalty terms to avoid over segmentation. Therefore, Formula (5) needs to be solved.

(5) $$\begin{array}{c} \underset{M,t_{m}}{\text{min}}\sum_{m=0}^{M}WSS_{t_{m},t_{m+1}}+C\cdot g\left(M,N\right), \end{array}$$

where *M* is the number of change points of input video segmentation; *t*_*m*_ is the position of the *m*-th change point. *t*_0_ and *t*_*M*+1_ are the beginning and end of the video respectively; *WSS*_*m*,*t*_*m*+1__ is the sum of squares within the group of the *m*-th segment in the embedded space. *g*(*M*, *N*)=*M*(ln(*N*/*M*)+1) is the standard punishment of Bayesian information, which is parameterized by the number of clips *M* and the number of scenes *N* in the videos. The *C* parameter is used to adjust the relative importance of the penalty. A higher penalty value of *C* would cause too many segments, so the choice of *C* value depends on the video. Adjust the *C* value by using a step size of 0.001, until the number of clusters is less than the number of scenes in the video.

The sum of the square distances between a set of points and its mean can be expressed as a function of the paired square distances between individual points. Therefore, the sum of squares within a group can be represented as scene segmentation.

(6) $$\begin{array}{c} {\text{WSS}}_{t_{m},t_{m+1}}≜\sum_{t=t_{m}}^{t_{m+1}^{-1}}\varphi \left(X_{t}\right)-\mu_{m}^{2}=\frac{1}{2\left(t_{m+1}-t_{m}\right)}\sum_{i,j=t_{m}}^{t_{m+1}^{-1}}\varphi \left(X_{i}\right)-\varphi {\left(X_{j}\right)}^{2}. \end{array}$$

Among them, *μ*_*m*_ is the average value of each scene shot feature.

(7) $$\begin{array}{c} \mu_{m}=\frac{1}{2\left(t_{m+1}-t_{m}\right)}\sum_{t=t_{m}}^{t_{m+1}^{-1}}\varphi \left(X_{t}\right). \end{array}$$

In this way, clustering targets can be minimized using dynamic programming methods. First, *WSS*_*r*,*r*+*d*_ is calculated for the starting point *r* and the duration *d* of each segment. Secondly, the optimal target values of *j* ∈ [1, *N*] lenses and *M* ∈ [0, *N* − 1] change points are calculated iteratively to minimize the target, as shown in equation (8), where *D*_0,*j*_=*WSS*_0,*j*_. In the end, the optimal number of variation points was chosen as *M*^*∗*^=$M*=\underset{M}{\arg\min}D_{M,N}+C\times g\left(M,N\right)$, and the optimal scene segmentation was reconstructed.

(8) $$\begin{array}{c} D_{M,j}=\underset{r=M,M+1,\cdots ,j-1}{\text{min}}\left(D_{M-1,r}+WSS_{r,j}\right). \end{array}$$

In the video scene segmentation algorithm, the input is the scene video stream, where the total number of video shots is *N* and the overall feature vector of the shot is *X*. The output is the scene boundary (lens number is *S*_*i*_). The specific algorithm steps are as follows:

1. The input video frequency stream is segmented into shots, and the key frame of the shot is identified by calculating the average distance of all frames within the shot. All shots and key frames after segmentation are numbered, namely, *S*_*i*_ and *S*_*f*_.
2. According to Formulas (1) and (2), the visual concept feature vector *v*(*s*) and text concept feature vector *t*(*s*) of the lens are extracted respectively. Then, all of its features are connected in series to obtain its global eigenvector *X*.
3. Adopt a deep network to learn an embedding function *φ*(*X*), embed video shot features in Euclidean space, and calculate the pair distance matrix between shots to get the similarity measure between shot features, and then get the semantic similarity between shots.
4. For each segment starting point *r* and segment duration *d*, calculate *WSS*_*r*,*r*+*d*°_.
5. Let *j* ∈ [1, *N*], *M* ∈ [0, *N* − 1], calculate an optimal target value containing *j* lenses and *M* change points to minimize the target *D*_*M*,*j*_. If *r* < *N*, continue to Step (5). While if *r*…*N*, go to Step (6).
6. The optimal number of change points *M*^*∗*^ is selected by calculation, and the lens number *S*_*i*_ of the corresponding scene boundary is output according to the location of the segmentation points, and the segmentation result of the video scene is finally obtained.

## 4. Experiment and Analysis

### 4.1. Model Training

The gradient descent method is used to optimize the algorithm, and the learning rate is taken as the default value of 0.01.  Table 1 shows the specific parameter configuration of network training.

**Figure 5:**
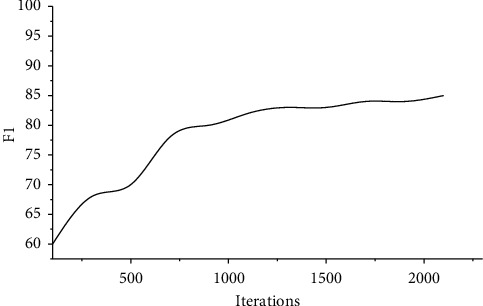
Results of model training.

Figure 5 shows the influence of the number of iterations on the final experimental results. It can be seen that when the number of iterations is small, the number of learning is not enough, so the accuracy of the final result is insufficient. However, because the visual features are only one part of the basis of video scene segmentation, the results still have a certain accuracy. Then, with the increase of iterations, the value of F increases, and after 2 000 iterations, it is stable.

### 4.2. Analysis of Model Validity

In order to verify the effectiveness of the proposed algorithm for video scene segmentation, five kinds of standard teaching videos are selected from the school online (https://www.icourse163.org/). The total length of the video is 128′19″, with 2760 lenses and 98 scenes. The details of the experiment are shown in Table 2.

**Figure 6:**
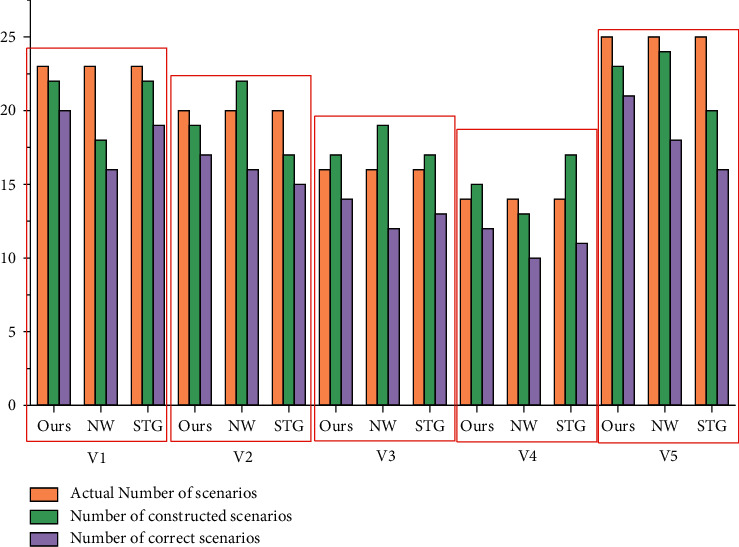
Comparison of scene construction.

**Figure 7:**
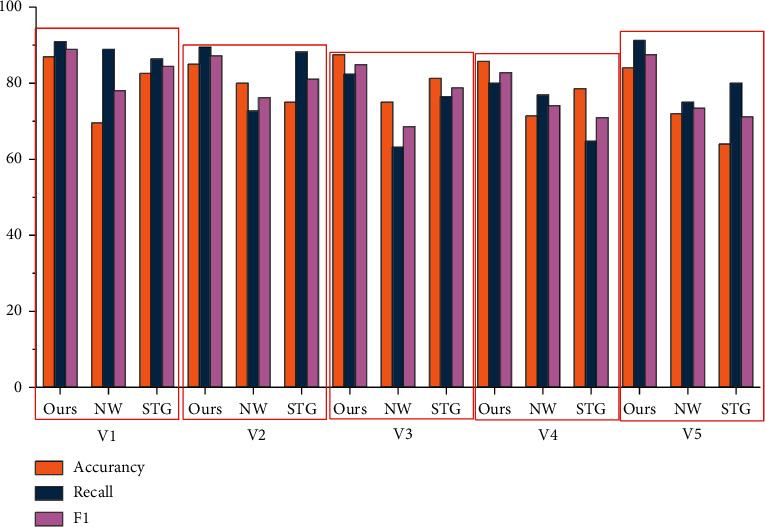
Comparison of model evaluation.

Recall, Precision, and F were used to evaluate the performance of the algorithm; the calculation formula of them are as follows:

(9) $$\begin{array}{c} \mathrm{Recall}=\frac{n_{c}}{n_{c}+n_{m}}\times 100\%=\frac{n_{c}}{n_{a}}\times 100\%,\\ \mathrm{Precision}=\frac{n_{c}}{n_{c}+n_{f}}\times 100\%=\frac{n_{c}}{n_{d}}\times 100\%,\\ F=\frac{2\times \mathrm{Precision}\times \mathrm{Recall}}{\mathrm{Precision}+\mathrm{Recall}}\times 100\%, \end{array}$$

where *n*_*c*_ is the total number of correct detected scenes; *n*_*m*_ is the total number of undetected scenarios; *n*_*f*_ is the total number of scenarios detected by errors; *n*_*a*_ is the total number of actual scenarios; *n*_*d*_ is the total number of detected scenarios. The proposed algorithm is compared with the NW algorithm [13] and STG algorithm [14], and the experimental results are shown in Figures 6 and 7.

From the data in the figure, we can see that the algorithm can construct video scene correctly. Compared with the NW algorithm and STG algorithm, the recall, precision, and F value of our algorithm are greatly improved. This is mainly because modesty only considers the combination of video underlying color features and NW algorithm, and the latter only integrates various visual and audio features in STG, the characteristics of temporal association and symbiosis between multiple modes in video data are not fully considered. We proposed a deep learning framework, where different low-level features from videos are extracted, and semantic concept features are combined with multi-modal semantic embedding space through triple deep network learning to segment videos into coherent scenes, thus effectively reducing the distance between low-level features and high-level semantics. Therefore, the scene segmentation effect achieved by our algorithm is better, and the algorithm is more universal for different types of videos.

### 4.3. System Testing

**Figure 8:**
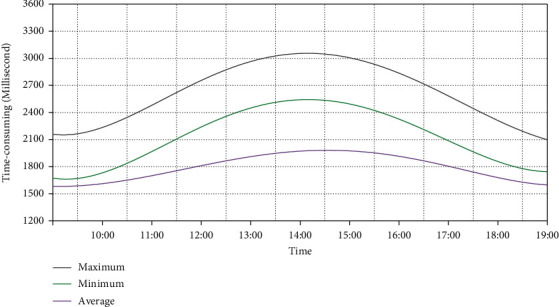
Response time of the system.

The response time of the system refers to the time spent by users when using the system. For the system, the response time is the time interval from clicking a page to displaying the page completely in the browser, which is divided into three parts, server response time, network response time, and client response time, respectively. The smaller the response time is, the faster the processing speed of the system is, and the shorter the waiting time of user operation is. Therefore, this paper tests the response time of the system, and the results are shown in Figure 8.

When users get a response between 2 and 5 seconds, the response speed of the system is considered to be good. When users get the response within 5 ∼ 8 seconds, the response speed of the system is considered to be very slow, but it is still acceptable. It can be seen from Figure 8 that the peak value of the maximum response time curve of the system is about 3 seconds, indicating that the response time of the platform is very fast. In addition, the peak value of the minimum response time curve is only 1.5 seconds, and the response time of the platform is better. The peak value of the average response time curve of the system is about 2.5 seconds, and the average response time of the system is less than 3 seconds, which meets the actual needs.

## 5. Conclusion

This paper realizes the characteristic application of EDIPT design thinking and business English in the crossing field. With business English video learning resources as the carrier, a business English learning system is constructed, which solves the problem of learning integration of business English learning groups and opens the exploration mode of design thinking in the field of education. In addition, aiming at the multimodal video scene segmentation algorithm based on a deep network, this paper extracts rich underlying features and semantic concept features from each lens, which realizes fast segmentation of video scene and achieves good experimental results. Moreover, it solves the problem of the “semantic gap” between video low-level *e* features and high-level semantics by multimodal feature fusion, making video scene segmentation more accurate and universal. In the following work, we will pay more attention to the functional test of the system, such as taking the form of the questionnaire.

## Figures and Tables

**Table 1:** Parameters of model training.

Training set	Epoch	Batch-size	Loss function	Number of iterations	Learning framework

8000	2	32	Cross entropy	2000	Keras

**Table 2:** The details of the experiment.

Video clip	Duration	Number of lenses	Number of scenes
V1 Discovering business opportunities and establishment of a business	29′13″	390	23
V2 organizational structure & recruiting and training employees	292′21″	436	20
V3 employee motivation and corporate culture	30′06″	587	16
V4 production, product and marketing	27′19″	633	14
V5 financial management and financing	31′47″	714	25
Total	128′19″	2760	98

## Data Availability

The dataset can be accessed from the corresponding author upon request.

## References

[1] Xiao T. (2022). Problems and improvement measures in practical teaching of business English in Colleges and universities. *Journal of Heilongjiang teachers’ development college*.

[2] Zhou Q. (2022). Informatization teaching based on learning platform -- Taking Business English interpretation course as an example. *Campus English*.

[3] Lin L., Shen S. (2016). Concept connotation and training strategy of design thinking. *Modern distance education research*.

[4] dong K. (2020). *Research on Scratch Learning Activity Design in Primary School from the Perspective of Design Thinking*.

[5] Pelaez K. (2018). *Latent Class Analysis and Random forest Ensemble to Identify At-Risk Students in Higher Education*.

[6] Takeda H., Yoshida S., Muneyasu M. *Tag-based Video Retrieval with Social Tag Relevance Learning*.

[7] Zhang D. (2009). A comprehensive theoretical framework for multimodal discourse analysis. *China foreign languages*.

[8] Cross J. (2011). Comprehending news videotexts: the influence of the visual content. *Language, Learning and Technology*.

[9] Han B. (2001). Language testing, theory, practice and development. *Foreign Language Teaching and Research*.

[10] Qian M., Zhao B., Gao Y. Exploring the training path of design thinking of students in educational technology.

[11] Ge W., Bai H., Ma H. (2020). Design thinking into the design of mixed curriculum and teaching intervention effect. *Modern Educational Technology*.

[12] Huang Q., Feng H., Liu Li (2022). Multimodal video scene segmentation optimization algorithm based on convolutional neural network. *Computer application research*.

[13] Chasanis V., Likas A., Galatsanos N. (2009). Scene detection in videos using shot clustering and sequence alignment. *IEEE Transactions on Multimedia*.

[14] Sidiropoulos P., Mezaris V., Kompatsiaris I., Meinedo H., Bugalho M., Trancoso I. (2011). Temporal video segmentation to scenes using high-level audiovisual features. *IEEE Transactions on Circuits and Systems for Video Technology*.

